# Genetic Diversity of *Stenotrophomonas maltophilia* and Clonal Transmission (ST92) in Critical Care Units at Hospital Juárez de México: MLST and Virulence Profiling

**DOI:** 10.3390/pathogens14111125

**Published:** 2025-11-05

**Authors:** Liliana Nicolas-Sayago, Clemente Cruz-Cruz, Emilio M. Durán-Manuel, Graciela Castro-Escarpulli, María G. Ortíz-López, Carlos A. Jiménez-Zamarripa, Araceli Rojas-Bernabé, Nayeli G. Nieto-Velázquez, Eduardo Tolentino-Sánchez, Juan C. Bravata-Alcántara, Julio C. Castañeda-Ortega, Benito Hernández-Castellanos, Adolfo López-Ornelas, Laura M. Márquez-Valdelamar, Dulce M. Razo Blanco-Hernández, Jonathan Puente-Rivera, Claudia C. Calzada-Mendoza, Yahaira de J. Tamayo-Ordóñez, María C. Tamayo-Ordóñez, Francisco A. Tamayo-Ordóñez, Miguel Á. Loyola-Cruz, Juan M. Bello-López

**Affiliations:** 1 Hospital Juárez de México, Mexico City 07760, Mexico; lilianaqbp0410@gmail.com (L.N.-S.); vaio_df@hotmail.com (J.C.B.-A.);; 2 Escuela Nacional de Ciencias Biológicas, Instituto Politécnico Nacional, Mexico City 11340, Mexico; 3 Sección de Estudios de Posgrado e Investigación, Escuela Superior de Medicina, Instituto Politécnico Nacional, Mexico City 11340, Mexico; 4 Hospital Psiquiátrico Dr. Samuel Ramírez Moreno, Valle de Chalco 56619, Mexico; 5 Facultad de Biología, Universidad Veracruzana, Xalapa 91090, Mexico; 6 Laboratorio de Secuenciación Genómica, LaNaBio, Instituto de Biología, Universidad Nacional Autónoma de México (UNAM), Mexico City 04510, Mexico; 7 Centro de Biotecnología Genómica, Instituto Politécnico Nacional, Reynosa 88710, Mexico; 8 Facultad de Ciencias Químicas, Universidad Autónoma de Coahuila, Saltillo 25280, Mexico; 9 Facultad de Química, Universidad Autónoma del Carmen, Ciudad del Carmen 24180, Mexico

**Keywords:** *Stenotrophomonas maltophilia*, multidrug resistance, biofilm formation, molecular typing, healthcare-associated infections

## Abstract

*Stenotrophomonas maltophilia* is considered one of the emerging bacterial agents causing healthcare-associated infections (HAIs) in hospital environments. This microorganism has been identified as multidrug-resistant, capable of forming mature biofilms—an ability that promotes adherence to surfaces and invasive medical devices, favoring persistence in hospital environments and the potential to generate outbreaks. The aim of this study was to characterize *S. maltophilia* strains isolated from HAI cases at the Hospital Juárez de México and to determine the presence of hidden outbreaks. Antibiotic resistance profiles were determined, along with the typing of 20 genes associated with virulence factors and the assessment of the ability to form mature biofilms on inert surfaces. Finally, sequence type (ST) was obtained through multilocus sequence typing (MLST) analysis, and a phylogenetic tree was constructed to determine the clonal diversity of the isolates. All strains showed uniform resistance to β-lactam antibiotics tested while remaining sensitive to fluoroquinolones, phenicols, tetracyclines, and trimethoprim/sulfamethoxazole. Some isolates exhibited adherent activity, with the “strong biofilm-former” phenotype predominating. Sixteen virulence-related genes were heterogeneously detected, revealing broad genetic diversity. MLST analysis grouped the isolates into nine ST related to infection cases reported in others countries. Phylogenetic analyses demonstrated the presence of three potential clones distributed across Internal Medicine and the Pediatric Intensive Care Unit. These results highlight the importance of investigating *S. maltophilia* as an HAI-associated pathogen that remains understudied.

## 1. Introduction

*Stenotrophomonas maltophilia* (formerly known as *Bacterium bookeri*) is a non-fermenting, aerobic, Gram-negative bacillus widely recognized as an opportunistic pathogen associated with healthcare-associated infections [1]. In 1983, it was reclassified within the genus *Xanthomonas*, and in 1993 it was finally assigned to *Stenotrophomonas* [2]. This bacterium has gained importance as a causal agent of healthcare-associated infections (HAI) worldwide, even though it was long considered merely a colonizer with limited pathogenic potential [3,4,5]. Reports indicate that the prevalence and mortality of infections caused by this microorganism have increased, with comorbidities being a major factor predisposing patients to infection [5,6,7,8].

This microorganism has been isolated from various environmental and hospital sources, including water, animals, soil, soaps, cleaning solutions, contact lenses, catheters, and even the hands of healthcare personnel [9,10,11,12,13,14]. Within the genus *Stenotrophomonas*, *S. maltophilia* is the only species capable of causing infections in humans, particularly among immunocompromised or hospitalized patients, especially those in intensive care units (ICU) [15,16]. Another risk group includes newborns, for whom low birth weight, small size for gestational age, and exposure to invasive procedures are considered predisposing factors for HAIs. Because neonatal patients have underdeveloped immune systems, *S. maltophilia* infections are potentially life-threatening in this population. In Mexico, this microorganism has been linked to outbreaks among neonates and isolated infection cases [10,17,18,19]. Infections caused by this microorganism may occur as part of polymicrobial infections (33–70%), and a synergistic interaction between *Pseudomonas aeruginosa* and *S. maltophilia* has been reported in mature biofilm formation in lungs, which is associated with increased mortality [15,20].

In Latin America and Europe, it is among the top ten pathogens causing pneumonia, while in Asia and the Pacific, it is one of the top four pathogens isolated from intra-abdominal infections. Additionally, resistance to carbapenems has been reported, complicating clinical management due to reduced therapeutic options [15]. Although *S. maltophilia* has been considered a bacterium with limited virulence, it possesses multiple pathogenicity factors such as pili, flagella, fimbrial structures, and adhesins that enable adherence/colonization of biotic and abiotic surfaces; lipopolysaccharides (LPS); and diffusible signal factors (DSF), which play a crucial role in regulating extracellular enzyme production (proteases, lipases, esterases, and fibrinolysins), microcolony formation, and tolerance to antibiotics and heavy metals [11,15,21,22].

*Stenotrophomonas maltophilia* exhibits resistance to various broad-spectrum antibiotics, including β-lactams, macrolides, fluoroquinolones, aminoglycosides, and carbapenems, limiting therapeutic options. Moreover, it can acquire mobile genetic elements and develop mutations in chromosomal regulators that induce efflux pump overexpression, resulting in multidrug-resistant phenotypes [23,24,25]. Transient phenotypic resistance, induced by environmental factors, also compromises treatment efficacy. In this scenario, the use of new antimicrobials, combination therapies, and efflux pump inhibitors has been proposed. Given the projected rise in *S. maltophilia* infections in vulnerable populations, urgent implementation of hospital control strategies and rational antibiotic use is required [26].

The ESKAPE group: *Enterococcus faecium*, *Staphylococcus aureus*, *Klebsiella pneumoniae*, *Acinetobacter baumannii*, *Pseudomonas aeruginosa*, and *Enterobacter* spp. represents the most clinically relevant multidrug-resistant pathogens responsible for the majority of healthcare-associated infections (HAIs) worldwide [27]. Although *S. maltophilia* is not traditionally included in this group, its increasing multidrug resistance and persistence in hospital environments have led several authors to recognize it as an “ESKAPE-like” pathogen. The need for this study arose from the growing detection of *S. maltophilia* at the Hospital Juárez de México (HJM) during the second wave of the COVID-19 pandemic (February–September 2021), a period characterized by a surge in mechanical ventilation and extended ICU stays that favored nosocomial transmission. Understanding its local epidemiology under these pandemic conditions was essential to assess its clinical impact and potential inclusion within the ESKAPE spectrum.

At the HJM, *S. maltophilia* isolation has historically been infrequent; however, its recent recurrent identification has raised interest in characterizing it to evaluate its pathogenic potential and clinical significance. In this context, *S. maltophilia* can be regarded as an emerging opportunistic pathogen with ESKAPE-like resistance traits rather than a classical member of the group [28,29]. The aim of this work was to characterize phenotypically and genetically *S. maltophilia* strains isolated from HAIs, providing evidence supporting its recognition as an emerging pathogen of local clinical relevance.

## 2. Materials and Methods

### 2.1. Stenotrophomonas maltophilia Strains and Biochemical Identification

*Stenotrophomonas maltophilia* strains (*n* = 11) were isolated from non-repetitive patients with confirmed HAIs (without prior antimicrobial treatment) at HJM, during February–September 2021, corresponding to the second wave of the COVID-19 pandemic. The strains were isolated from two clinical sources: pulmonary aspirate (*n* = 9) and blood (*n* = 2). The patient care areas where *S. maltophilia* infections occurred were: Pediatric Intensive Care Unit (PICU), Internal Medicine (IM), and wards designated for patients with severe COVID-19. Bacterial isolation was performed using standard culture protocols in selective and differential media for Gram-negative bacilli (MacConkey agar). Identification was carried out to the genus and species levels using the automated Vitek 2-XL system (bioMérieux, Durham, NC, USA), following the manufacturer’s instructions.

### 2.2. Genetic Confirmation of Stenotrophomonas maltophilia Strains by 16S rRNA Sequencing

For molecular assays, total DNA was extracted and purified using the DNeasy Blood & Tissue Kit (QIAGEN, Venlo, The Netherlands), following the manufacturer’s instructions. The integrity of genomic DNA was verified on horizontal 0.8% agarose gels and used as template in endpoint PCR assays. Amplifications were performed in a T100 Thermal Cycler (Bio-Rad Laboratories, Hercules, CA, USA). PCR reactions targeting the full-length *16S rRNA* gene were performed with universal primers 27F (5′-AGAGTTTGATCMTGGCTCAG-3′) and 1492R (5′-TACGGYTACCTTGTTACGACTT-3′), using the conditions described by DeSantis et al. (2007) [30]. Cycling conditions were as follows: pre-denaturation at 95 °C for 5 min, denaturation at 95 °C for 30 s, annealing at 57 °C for 40 s, and extension at 72 °C for 1 min, with a final extension at 72 °C for 7 min at the end for 30 cycles.

Amplicons were visualized on 1.5% agarose gels using 1 × Tris-Borate-EDTA (TBE) buffer. PCR products were purified using the QIAquick PCR Purification Kit (QIAGEN, Hilden, Germany) according to the manufacturer’s instructions and sequenced at the Instituto de Biología, Universidad Nacional Autónoma de México (UNAM), using a DNA Analyzer 3730 × L (Applied Biosystems, Foster City, CA, USA). Sequences were compared to the GenBank database via the BLAST algorithm (version 2.14.1+) (http://blast.ncbi.nlm.nih.gov) (accessed on 10 August 2025), using coverage (>80%) and identity (>90%) thresholds.

### 2.3. Antimicrobial Susceptibility and Resistance Profiles of Stenotrophomonas maltophilia Strains

Antimicrobial susceptibility testing was performed by the broth microdilution method using Mueller–Hinton (MH) broth, according to CLSI (2024) standards [31]. In 96-well flat-bottom polystyrene plates, 100 µL of MH broth containing antibiotic concentrations of ceftazidime (4–64 µg/mL), levofloxacin (2–64 µg/mL), chloramphenicol (4–64 µg/mL), trimethoprim–sulfamethoxazole (2–32 µg/mL), and ticarcillin–clavulanate (8–128 µg/mL) were inoculated with 10 µL of bacterial suspensions adjusted to 0.5 McFarland turbidity (in triplicate). Plates were incubated at 35 ± 2 °C for 18–24 h, and the minimum inhibitory concentration (MIC) was interpreted to categorize isolates as susceptible, intermediate, or resistant. *Staphylococcus aureus* ATCC 25923 [32], *E. coli* ATCC 25922 [33], and *P. aeruginosa* ATCC 27,853 [34] were used as internal quality controls.

### 2.4. In Vitro Formation of Mature Biofilms of Stenotrophomonas maltophilia Strains

Biofilm formation ability was evaluated as described by Qi et al. (2016) and Cruz-Córdova et al. (2020), with minor modifications [18,35]. Briefly, strains were cultured overnight in 3 mL of LB broth at 37 °C with shaking, then subcultured again in fresh LB broth. Cultures were centrifuged, and bacterial pellets were resuspended in cold isotonic saline and adjusted to 0.5 McFarland. Aliquots (150 µL) of fresh LB broth were dispensed into wells of sterile 96-well flat-bottom microplates, and 50 µL of bacterial suspension was added (in triplicate).

Plates were sealed and incubated under aerobic conditions at 37 °C for 48 h. After incubation, wells were washed three times with sterile 1 × PBS (pH 7.4), stained with 0.1% crystal violet (200 µL) for 30 min at room temperature, rinsed again, and biofilm-bound dye was solubilized with 30% acetic acid. Optical density (OD_600_) was measured using a spectrophotometer (Epoch, BioTek Instruments, Winooski, VT, USA). Uninoculated wells containing only LB served as negative controls.

### 2.5. Virutyping of Stenotrophomonas maltophilia Strains

Endpoint PCR assays targeting 20 virulence and adherence-associated genes were performed according to Cruz-Córdova et al. (2020) [18]. Screened genes included *lktD* (61 °C), *gspD* (53 °C), *virB* (61 °C), *hcp* (60 °C), *entA* (61 °C), *tpsB* (62 °C), *stmPr1* (61 °C), *rmlA* (55 °C), *hlyIII* (61 °C), *hgbB* (60 °C), *zot* (62 °C), *frpC* (61 °C), *pilU* (61 °C), *fliC* (61 °C), *afaD* (62 °C), *papD* (61 °C), *motA* (61 °C), *fhaB* (61 °C), and *fimH* (61 °C). PCR reactions were performed out under standard cycling conditions consisting of an initial denaturation at 95 °C for 5 min; followed by 30 cycles of denaturation at 95 °C for 30 s, annealing for 40 s at the corresponding temperature for each gene as indicated above, and extension at 72 °C for 1 min; with a final extension at 72 °C for 7 min. Amplicons were resolved in 2% agarose gels in 1 × TAE buffer at 65 V for 2 h and visualized under UV light. Randomly selected amplicons were purified and sequenced to confirm their identity.

### 2.6. Multilocus Sequence Typing and Phylogenetic Analysis

MLST was performed as described by Kaiser et al. (2009), Jolley et al. (2018), and Cruz-Córdova et al. (2020) [18,36,37]. Seven housekeeping genes *atpD* (61 °C), *gapA* (64 °C), *guaA* (60 °C), *mutM* (55 °C), *nuoD* (55 °C), *ppsA* (61 °C), and *recA* (61 °C) were amplified and sequenced according to the Oxford University MLST scheme. PCR cycling conditions consisted of an initial denaturation at 95 °C for 5 min, followed by 30 cycles of denaturation at 95 °C for 30 s, annealing for 40 s at the specific temperature indicated for each gene, and extension at 72 °C for 1 min, with a final extension at 72 °C for 7 min. Amplicons were visualized on 2% agarose gels in 1 × TAE buffer at 65 V for 2 h under UV illumination. Purified PCR products were sequenced in both directions, and allelic profiles and sequence types (ST) were determined using the PubMLST platform (https://pubmlst.org/smaltophilia/) (accessed on 10 August 2025). To assess clonal relationships, sequences of the seven MLST housekeeping genes were aligned using MAFFT v7 (https://mafft.cbrc.jp/alignment/software/) (accessed on 12 July 2025). Concatenated alignments were used to construct a phylogenetic tree by the maximum likelihood method with 100 bootstrap replicates [38,39].

## 3. Results

### 3.1. Epidemiological Distribution of Stenotrophomonas maltophilia Isolates

During the study period (February–September 2021), corresponding to the second wave of the COVID-19 pandemic, eleven *S. maltophilia* isolates associated with HAIs were identified across different clinical areas of HJM.

**Figure 1:**
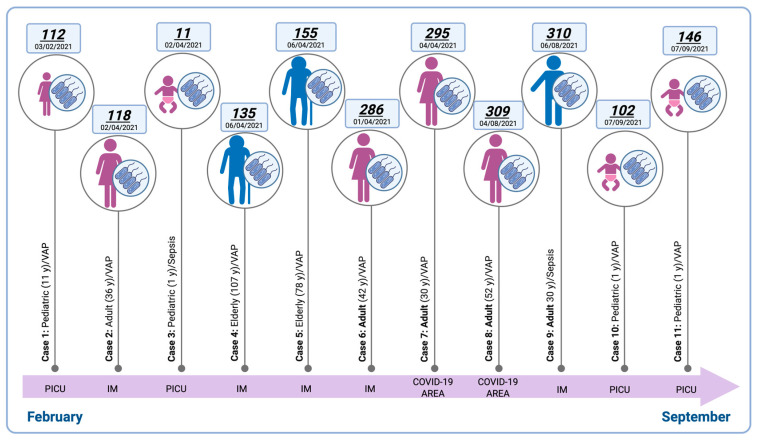
Temporal distribution and demographic characteristics of HAI cases with *Stenotrophomonas maltophilia* isolation at Hospital Juárez de México, February–September 2021. M: Internal Medicine; PICU: Pediatric Intensive Care Unit, VAP: Ventilator Associated Pneumonia. Bold labels represent chronological HAI cases during the study period. Created in BioRender. Nolasco, A. (2025) https://BioRender.com/iat9gc1 (accessed on 15 July 2025).

The temporal distribution showed sustained recurrence, with the highest concentration of cases observed in April *(n* = 4, Internal Medicine), followed by PICU (*n* = 3), and COVID-19 wards (*n* = 2). Most cases were associated with ventilator-associated pneumonia (VAP, *n* = 2), while fewer involved sepsis in pediatric and adult patients (*n* = 2). Patients ranged in age from one year to over one hundred years, underscoring the opportunistic nature of *S. maltophilia* across diverse age groups and clinical contexts. Chronological and spatial distribution (Figure 1) suggested possible undetected transmission events or outbreaks, particularly in IM and PICU, where temporal clustering of cases was observed. 

### 3.2. Phenotypic Antimicrobial Resistance Profiles

**Figure 2:**
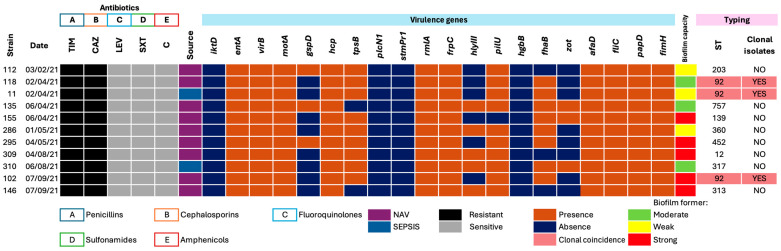
Phenotypic and genotypic profiles of *Stenotrophomonas maltophilia* strains isolated from HAI cases at Hospital Juárez de México, February–September 2021. Antibiotics: TIM: ticarcillin–clavulanate, CAZ: ceftazidime, LEV: levofloxacin, SXT: trimethoprim–sulfamethoxazole, and C: chloramphenicol.

The antimicrobial resistance profile (Figure 2) revealed that all strains were resistant to the β-lactam antibiotics tested, including ticarcillin–clavulanate (TIM) and ceftazidime (CAZ). In contrast, all isolates were susceptible to fluoroquinolones, chloramphenicol, and trimethoprim-sulfamethoxazole. This phenotype suggests intrinsic β-lactam resistance, consistent with multidrug-resistant (MDR) behavior.

### 3.3. Distribution of Virulence Genes

As shown in Figure 2, all eleven isolates displayed broad genotypic diversity, with detection of 16 out of 20 virulence-associated genes. Genes linked to secretion systems (*gspD*, *virB*, *hcp*) were highly prevalent, indicating conserved mechanisms for effector protein export. Similarly, *tpsB* (type V cytolysin activator) and *entA* were detected in most isolates, suggesting potential for tissue damage and colonization. Determinants associated with LPS biosynthesis (*rmlA*) and hemolysin synthesis (*hlyIII*) were detected in 100% and 45% of isolates, respectively.

Adhesion and motility genes (*pilU*, *fliC*, *afaD*, *fimH*, *papD*, *motA*, *fhaB*) were consistently present, correlating with strong or moderate biofilm-forming phenotypes. Toxin-associated gene *zot* occurred at low frequency, whereas *frpC* (RTX toxin) was detected in all isolates (100%). Overall, genotypic patterns reflected predominance of secretion and adherence factors, consistent with strong biofilm formation capacity, while toxigenic determinants appeared less frequently.

### 3.4. Clonal Typing and Relationships Among Isolates

As shown in Figure 2, three isolates were assigned to ST92, mainly distributed in Internal Medicine and PICU, suggesting the circulation of an epidemic clone. The remaining isolates were classified as unique clones, indicating considerable genetic diversity within the hospital and ruling out a single transmission event. However, the recurrence of ST92 across different months supports the hypothesis of clonal outbreaks.

### 3.5. Phylogenetic Analysis of Stenotrophomonas maltophilia Strains

**Figure 3:**
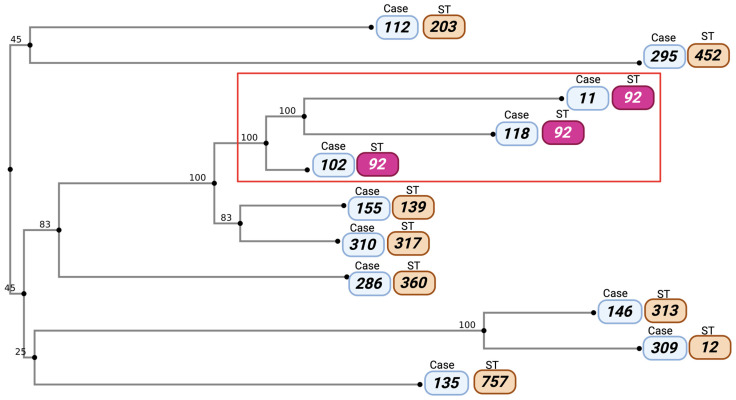
Phylogenetic tree of *Stenotrophomonas maltophilia* obtained through multilocus sequence typing (MLST) based on concatenated *atpD*, *gapA*, *guaA*, *mutM*, *nuoD*, *ppsA*, and *recA* genes. The red box highlights the cluster corresponding to clone ST92 (purple colour) (isolates 11, 118, and 102). Created in BioRender. Nolasco, A. (2025) https://BioRender.com/1xg5x30 (accessed on 15 July 2025).

As shown in Figure 3, phylogenetic analysis based on concatenated sequences of seven housekeeping genes (*atpD*, *gapA*, *guaA*, *mutM*, *nuoD*, *ppsA*, and *recA*) revealed high clonal diversity among the eleven clinical isolates. MLST results identified nine distinct sequence types (STs), reflecting a heterogeneous population structure within the hospital. ST92, detected in three isolates (strains 11, 118, and 102), formed a well-defined clade (highlighted in the red box), suggesting possible clonal transmission or the circulation of an epidemic lineage in critical hospital areas. This group shared genetic proximity and temporal–spatial overlap, being isolated in April and September, mainly from PICU and IM (Figure 1 and Figure 2). Other isolates grouped into independent phylogenetic branches corresponding to ST203, ST452, ST139, ST317, ST360, ST313, ST12, and ST757, indicating the coexistence of unrelated unique isolates. Consistently, strains within this clone shared uniform β-lactam resistance phenotypes and the presence of secretion system genes (T2SS, T4SS, T6SS), reinforcing their potential role as high-risk clones.

## 4. Discussion

Surveillance of HAIs often prioritizes classical ESKAPE pathogens; however, *S. maltophilia* has emerged as a “non-traditional” but increasingly relevant agent, especially in critical-care settings. Its environmental ecology, intrinsic resistance, and biofilm-forming ability promote persistence and may be underestimated when surveillance focuses exclusively on conventional ESKAPE members [29,40,41]. In our hospital, the typical ESKAPE pathogens historically predominate, *S. maltophilia* had not previously been considered as priority pathogen [42,43,44,45,46]. The findings of this study confirm that *S. maltophilia* represents an emerging opportunistic pathogen of growing clinical and epidemiological importance at HJM. This observation supports previous reports that classify *S. maltophilia* as an emerging multidrug-resistant pathogen exhibiting ESKAPE-like behavior and clinical significance [28,29].

Molecular confirmation of eleven clinical isolates from critical areas revealed notable clonal diversity, with coexistence of unrelated strains alongside a defined clonal cluster (ST92). This clone, identified in isolates from PICU and IM, suggests a nosocomial transmission event. ST92 has been previously reported in clinical and environmental isolates from Iran [47]. Temporal and spatial overlap observed in our hospital supports the hypothesis of an epidemic clone, whereas other isolates with unique STs likely reflect independent introductions linked to environmental reservoirs or colonized patients. ST155, widely reported in Italy, Poland, and Iran in patients with chronic diseases and hospital-acquired infections, was also detected [48,49,50].

Phenotypically, all isolates exhibited uniform resistance to β-lactams (ceftazidime and ticarcillin–clavulanate), consistent with intrinsic resistance mediated by chromosomal β-lactamases L1 and L2 [51]. Conserved susceptibility to fluoroquinolones, chloramphenicol, tetracyclines, and trimethoprim–sulfamethoxazole (TMP-SMX) confirms the continued therapeutic value of these agents, particularly TMP-SMX, the first-line treatment [15,52]. Nevertheless, TMP-SMX-resistant strains have been documented [40], emphasizing the need for continuous phenotypic and genotypic surveillance to guide rational therapy and avoid empirical use.

Regarding virulence genotypes, secretion system determinants predominated—type II (*gspD*), type IV (*virB*), and type VI (*hcp*)—facilitating effector protein export and hydrolytic enzyme production [53]. Universal detection of *entA* suggests an adaptive advantage in iron acquisition and proliferation [54,55]. Additionally, *tpsB* confers potential for tissue invasion [56], and *rmlA*, involved in LPS biosynthesis, was present in all isolates, contributing to cell envelope integrity and biofilm formation.

Adhesion and motility genes (*pilU*, *fliC*, *fimH*, *papD*, *motA*, *fhaB*) were associated with adherence and motility, explaining the “strong biofilm-former” phenotype [18,41]. This phenotype has been previously documented in clinical strains reinforcing the role of *S. maltophilia* in surface and device persistence [57]. Biofilm formation in all isolates is highly relevant due to its role in environmental persistence, antimicrobial tolerance, and nosocomial dissemination. This finding is especially important during the COVID-19 pandemic, when mechanical ventilation demands increased exposure and colonization risks. The identification of biofilm-forming strains supports the hypothesis of surface-associated transmission via contaminated medical equipment. These findings strengthen the proposal to consider *S. maltophilia* as a functional ESKAPE member, given its phenotypic and genetic characteristics. The ESKAPE acronym-encompassing *E. faecium*, *S. aureus*, *K. pneumoniae*, *A. baumannii*, *P. aeruginosa*, and *Enterobacter* spp.—has been revised by several authors to include emerging pathogens such as *S. maltophilia* [58].

## 5. Conclusions

The findings of this study demonstrate that *S. maltophilia* isolates from HAI at the HJM exhibit significant genetic diversity but share key phenotypic characteristics, including uniform resistance to β-lactam antibiotics (ceftazidime and ticarcillin–clavulanate) and preserved susceptibility to fluoroquinolones, chloramphenicol, and trimethoprim-sulfamethoxazole. All isolates displayed the capacity to form mature biofilms and harbored virulence determinants associated with secretion systems (T2SS, T4SS, T6SS) and adhesion, underscoring their environmental persistence and potential for nosocomial transmission. The identification of a clonal lineage (ST92) circulating within critical care units during the second wave of the COVID-19 pandemic links these findings directly to the local epidemiological context, where prolonged hospitalization and invasive procedures increased infection risk. These results emphasize the need for strengthened surveillance and infection control strategies targeting *S. maltophilia* and other “ESKAPE-like” pathogens in tertiary-care settings.

## Data Availability

The original contributions presented in this study are included in the article. Further inquiries can be directed to the corresponding author(s).
